# PGD2 displays distinct effects in diffuse large B-cell lymphoma depending on different concentrations

**DOI:** 10.1038/s41420-023-01311-6

**Published:** 2023-02-01

**Authors:** Shunfeng Hu, Tiange Lu, Juanjuan Shang, Yiqing Cai, Mengfei Ding, Xiangxiang Zhou, Xin Wang

**Affiliations:** 1 Department of Hematology, Shandong Provincial Hospital, Shandong University, Jinan, Shandong 250021 China; 2 Department of Hematology, Shandong Provincial Hospital Affiliated to Shandong First Medical University, Jinan, Shandong 250021 China; 3 Branch of National Clinical Research Center for Hematologic Diseases, Jinan, Shandong 250021 China; 4 National Clinical Research Center for Hematologic Diseases, The First Affiliated Hospital of Soochow University, Suzhou, 251006 China

**Keywords:** Drug development, Cell growth, DNA damage response, Prognostic markers, Apoptosis

## Abstract

Prostaglandin D2 (PGD2), an arachidonic acid metabolite, has been implicated in allergic responses, parasitic infection and tumor development. The biological functions and molecular mechanisms of PGD2 in diffuse large B-cell lymphoma (DLBCL) are still undefined. In this study, we firstly found the high concentration of serum PGD2 and low expression of PGD2 receptor CRTH2 in DLBCL, which were associated with clinical features and prognosis of DLBCL patients. Interestingly, different concentration of PGD2 displayed divergent effects on DLBCL progression. Low-concentration PGD2 promoted cell growth through binding to CRTH2 while high-concentration PGD2 inhibited it via regulating cell proliferation, apoptosis, cell cycle, and invasion. Besides, high-concentration PGD2 could induce ROS-mediated DNA damage and enhance the cytotoxicity of adriamycin, bendamustine and venetoclax. Furthermore, HDAC inhibitors, vorinostat (SAHA) and panobinostat (LBH589) regulated CRTH2 expression and PGD2 production, and CRTH2 inhibitor AZD1981 and high-concentration PGD2 enhanced their anti-tumor effects in DLBCL. Altogether, our findings demonstrated PGD2 and CRTH2 as novel prognostic biomarkers and therapeutic targets in DLBCL, and highlighted the potency of high-concentration PGD2 as a promising therapeutic strategy for DLBCL patients.

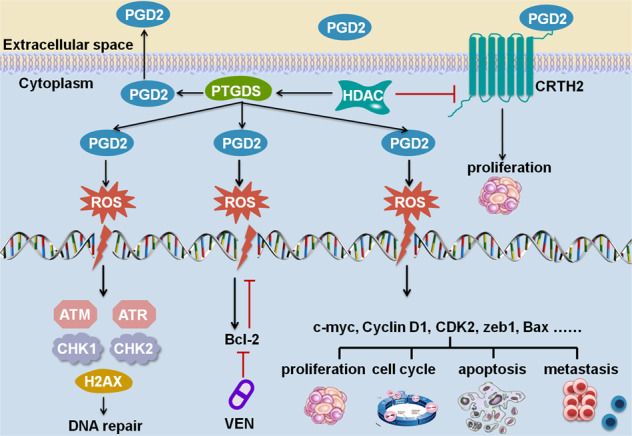

## Introduction

Diffuse large B-cell lymphoma (DLBCL), the most common type of non-Hodgkin lymphoma (NHL), is characterized by high morbidity and mortality in the world [1]. With the great advances in novel treatment regimens for DLBCL [2], such as targeted therapy, immunochemotherapy and epigenetic therapy, a large fraction of DLBCL patients could achieve encouraging prognosis. However, there are still 40–50% of DLBCL patients who are eventually refractory or relapsed and even die due to disease progression [3, 4]. Therefore, further investigations into novel therapeutic strategies are required for DLBCL treatment [5].

Prostaglandins are synthesized by cyclooxygenase (COX) and prostaglandin synthase from cell membrane-derived arachidonic acid. Current researches of prostaglandins have been mainly focused on allergic response, cardiovascular disease, and sleep promotion [6, 7]. Prostaglandins could be divided into multiple types (*A*–*I*) according to different structures. Among them, PGE2 has been found to promote tumor progression [8, 9] whereas PGD2 exerts as anti-proliferative factor in various cancers, including hematological malignancy [10–12]. Currently, two distinct types of PGD2 synthase have been identified: the hematopoietic type (H-PGDS) and the lipocalin type (L-PGDS, also called PTGDS). Different from the stable expression of H-PGDS in vivo, the expression level of PTGDS could be significantly influenced by host status, and our previous research has demonstrated the increased expression of PTGDS in the tissue and serum of DLBCL patients [13]. Besides, there are two kinds of PGD2 receptors in human, DP1 and DP2, and DP2 has been referred as chemo-attractant receptor molecule expressed on Th2 (CRTH2) due to its ability of mediating chemotactic responses of type 2 helper T lymphocytes to PGD2. Recent studies showed that PGD2 has been involved in tumor development via multiple mechanisms, including receptor-mediated response, peroxisome proliferator-activated receptor γ (PPARγ) [10, 14], and reactive oxygen species (ROS) production [15, 16]. However, the biological functions and molecular mechanisms of PGD2 in DLBCL have not been reported yet.

Epigenetic modification is the regulation of gene expression without changes in DNA sequence, including DNA methylation, histone modifications, non-coding RNAs and so on [17]. Among them, histone acetylation modification [18] has been involved in the development of multiple diseases, especially in tumor development [19]. The dynamic equilibrium of histone acetylation is controlled by histone deacetylases (HDACs) and histone acetyl transferases (HATs) [20]. Besides, abnormal expression of HDACs has been found in hematological cancers, including DLBCL [21–23], and several HDAC inhibitors, such as vorinostat (chemically named SAHA), romidepsin, panobinostat (also called LBH589) and belinostat, have been approved for the clinical treatment of hematologic cancers [24]. Furthermore, recent study found that HDAC inhibitors could regulate the release of prostaglandins in microglia, including PGD2 and PGE2 [25]. Yet, whether and how HDAC inhibitors are associated with PGD2 effects in DLBCL needs to be further investigated.

Herein, our present study aimed to explore the expression level, regulatory effects and molecule mechanisms of PGD2 in DLBCL. Increased concentration of serum PGD2 and decreased expression of CRTH2 were found in DLBCL patients, both of which were correlated with clinical characteristics and prognosis. Interestingly, different concentration of PGD2 displayed divergent effects on DLBCL progression. Moreover, HDAC inhibitors could regulate CRTH2 expression and PGD2 production, and CRTH2 inhibitor AZD1981 and high-concentration PGD2 enhanced their anti-tumor effects in DLBCL. Altogether, our findings found the distinct effects and underlying mechanisms of PGD2 with different concentration in DLBCL, and provided novel biomarkers and therapeutic targets for DLBCL treatment.

## Results

### Elevated serum PGD2 and decreased expression of CRTH2 were correlated with DLBCL progression

**Fig. 1:**
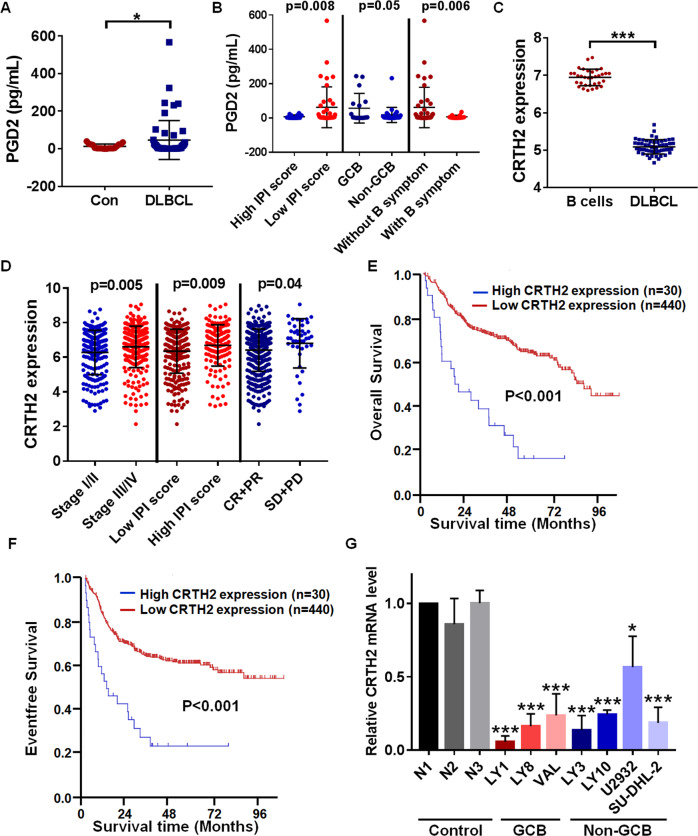
Elevated serum PGD2 and decreased expression of CRTH2 were correlated with DLBCL progression. **A** The concentration of serum PGD2 was higher in DLBCL patients (*n* = 53) than healthy controls (*n* = 19). **B** We compared serum PGD2 concentration in DLBCL patients grouped according to IPI score, GCB subtype and B symptom, and found the association between serum PGD2 concentration and clinical features in DLBCL patients (*n* = 53). **C** Analysis of GSE56315 dataset showed the decreased mRNA expression level of CRTH2 in DLBCL patients. **D** To compare the expression of CRTH2 in DLBCL patients grouped according to stage, IPI score and therapeutic efficacy, analysis of GSE31312 dataset found the association between CRTH2 expression and clinical features in DLBCL patients. **E**, **F** Kaplan–Meier survival analysis showed the association between CRTH2 expression and OS and EFS in DLBCL patients, respectively. **G** RT-PCR results showed decreased expression of CRTH2 in DLBCL cell lines, including three GCB cell lines (LY1, LY8, VAL) and four non-GCB cell lines (LY3, LY10, U2932, and SU-DHL-2), in comparison with CD19^+^ B cells from PBMCs of three healthy donors (three time points). The expression of CRTH2 in healthy control (N1) was adjusted to 1. Data are shown as the mean ± SD. *p < 0.05; **p < 0.01; ***p < 0.001. Abbreviations: DLBCL diffuse large B-cell lymphoma, GCB germinal center B-cell-like, IPI international prognostic index, CR complete remission, PR partial remission, SD stable disease, PD progressive disease.

To evaluate the expression level of PGD2 in DLBCL patients, analysis based on ELISA assay showed that compared with healthy control (*n* = 19), DLBCL patients (*n* = 53) displayed higher concentration of serum PGD2 (46.32 pg/mL vs 12.24 pg/mL) (Fig. 1A). Besides, there were ten DLBCL patients who had significantly high level of serum PGD2 (>70 pg/mL), and we further divided DLBCL patients into two subgroups according to the concentration of serum PGD2 (*n* = 10 vs 43, Supplementary Table 1). It is found that high concentration of serum PGD2 was statistically correlated with low International Prognostic Index (IPI) score, GCB subtype, B symptoms absence and low lymphocyte-to-monocyte ratio (LMR), indicating the association between PGD2 and DLBCL progression (Fig. 1B, Supplementary Table 2). However, there was no statistical correlation between serum PGD2 concentration and Ann Arbor stage, double expression, therapeutic efficacy and other clinical parameters in DLBCL patients (Supplementary Table 2), which required further large-sample exploration.

As PGD2 has participated in the development of several tumors through binding to its receptor, we further explored the expression and clinical association of CRTH2 in DLBCL. Analysis of GSE56315 dataset showed the decreased mRNA expression level of CRTH2 in DLBCL patients (Fig. 1C). Moreover, based on GSE31312, it’s found that low expression of CRTH2 was associated with stage I/II (*p* = 0.005), low IPI score (*p* = 0.009) and favorable therapeutic efficacy (*p* = 0.04) (Fig. 1D). Kaplan–Meier survival curve analysis showed that DLBCL patients with high CRTH2 expression displayed worse overall survival (OS) (p < 0.001, Fig. 1E) and event-free survival (EFS) (*p* < 0.001, Fig. 1F), suggesting the involvement of CRTH2 in DLBCL progression. Moreover, we further clarified the decreased expression of CRTH2 in DLBCL cell lines (*n* = 7) in comparison with CD19^+^ B cells from PBMCs of healthy donors (*n* = 3) (Fig. 1G), among them LY1 cells (GCB subtype) and LY3 cells (non-GCB subtype) were chosen for subsequent experiments according to CRTH2 expression level. Furthermore, the analysis based on Human Protein Atlas database showed the negative expression of CRTH2 protein in lymphoma tissue (*n* = 11) through immunohistochemistry (Supplementary Fig. S1). Taken together, our findings indicated PGD2 and CRTH2 as potential prognostic predictors in DLBCL patients.

### Different concentration of PGD2 displayed distinct effects on DLBCL cell proliferation

**Fig. 2:**
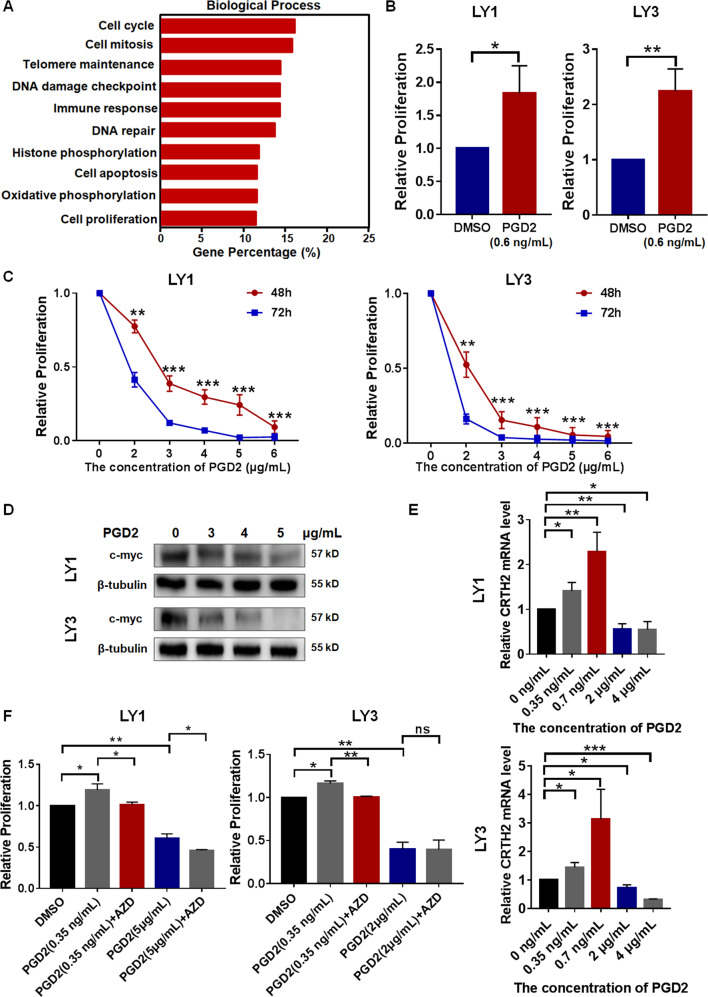
Different concentration of PGD2 displayed distinct effects on DLBCL cell proliferation. **A** GO analysis of CRTH2 associated genes (*p* < 0.05) based on GSE31312 database indicated the association between CRTH2 and tumor development. **B** The treatment with PGD2 (0.6 ng/mL), similar concentration to serum PGD2, promoted cell proliferation. **C**, **D** High-concentration PGD2 dose-dependently decreased cell proliferation and c-myc expression in DLBCL cells. **E** Low-concentration PGD2 increased the expression level of CRTH2 while high-concentration PGD2 significantly decreased it. The expression of CRTH2 in DLBCL cells without drug treatment was adjusted to 1. **F** AZD1981 (AZD), the CRTH2 inhibitor, rescued the pro-proliferation effects of low-concentration PGD2, but not the anti-proliferation effects of high-concentration PGD2 in DLBCL cells. DLBCL cells were treated with drugs for 48 h. Data are shown as the mean ± SD. *p < 0.05; **p < 0.01; ***p < 0.001.

To elucidate the role of PGD2 on DLBCL progression, we analyzed the data from GSE31312 and GSE57611 dataset, and found that CRTH2 were closely associated with biological processes involved in DLBCL progression, including cell proliferation, apoptosis, cell cycle, and migration (Fig. 2A, Supplementary Fig. S2). Therefore, we performed further experiments to validate the effects of PGD2 on DLBCL progression.

To explore the effects of PGD2 on cell proliferation, we performed CCK-8 assays with PGD2 range from 1 ng/mL to 10 μg/mL. It’s found that low-concentration PGD2 (1–10 ng/mL) could promote the proliferation of DLBCL cells and high-concentration PGD2 (1–10 μg/mL) significantly inhibited it (Supplementary Fig. S3). As the concentration of serum PGD2 in DLBCL patients reached about 0.6 ng/mL, further study showed that PGD2 (0.6 ng/mL) could promote the proliferation of DLBCL cells (Fig. 2B), indicating that serum PGD2 might promote the progression of DLBCL. Previous studies have found that high-concentration PGD2, reaching to 3.5 μg/mL, could inhibit the development of various tumors, including gastric cancer [10], prostate tumor [14], astrocytoma [26] and colon cancer [27]. Our study indicated that high-concentration PGD2 (2–6 μg/mL) displayed anti-proliferation effects in DLBCL cells in both time- and concentration-dependent manners (Fig. 2C). Since c-myc has been known for its role in cell proliferation, we found that high-concentration PGD2 decreased its expression in a concentration-dependent manner (Fig. 2D).

As the dependence of PGD2 effects on receptor-mediated response in tumor development, we performed further experiments to explore whether CRTH2 was involved in the effects of PGD2 in DLBCL. It’s found that low-concentration PGD2 could increase the expression level of CRTH2 while high-concentration PGD2 significantly decreased it (Fig. 2E). Moreover, AZD1981, the inhibitor of CRTH2, could rescue the pro-proliferation effects of low-concentration PGD2, but not the anti-proliferation effects of high-concentration PGD2 in DLBCL cells (Fig. 2F). These results indicated that low-concentration PGD2 increased cell proliferation mainly through binding to CRTH2, while CRTH2 did not play a major role in the anti-proliferation effects of high-concentration PGD2 in DLBCL cells.

### High-concentration PGD2 regulated the cell cycle, apoptosis and invasion of DLBCL cells

Apart from cell proliferation, analysis of GSE31312 and GSE57611 dataset indicated the association between CRTH2 and other biological functions in DLBCL, including cell cycle, apoptosis and cell invasion. Therefore, we performed further in vitro experiments to validate the association in DLBCL cells.

**Fig. 3:**
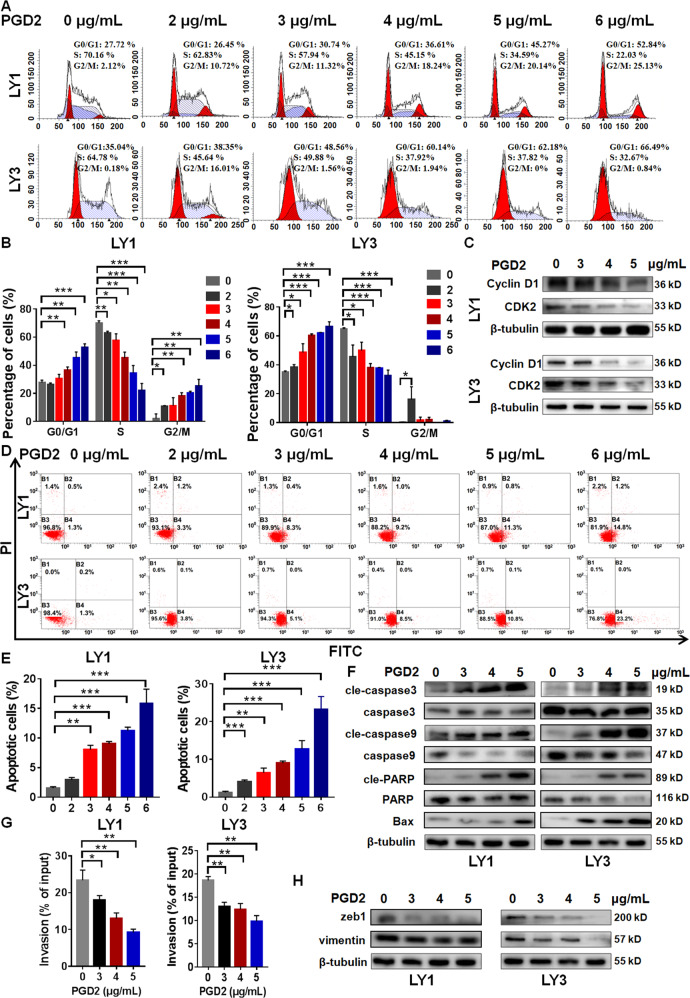
High-concentration PGD2 regulated the cell cycle, apoptosis, and invasion of DLBCL cells. After the treatment with high-concentration PGD2 for 48 h, **A**–**C** High-concentration PGD2 induced G0/G1 cell cycle arrest and decreased the expression of Cyclin D1 and CDK2 in DLBCL cells. **D**–**F** High-concentration PGD2 induced cell apoptosis (**D** representative images and **E** statistic results) and influenced the expression of apoptosis associated proteins. **G**, **H** High-concentration PGD2 suppressed cell invasion and the expression of zeb1 and vimentin. Data are shown as the mean ± SD. *p < 0.05; **p < 0.01; ***p < 0.001.

In our study, high-concentration PGD2 was found to induce cell cycle arrest at G0/G1 phase in a concentration dependent manner (Fig. 3A, B). Moreover, high-concentration PGD2 could significantly reduce the expression of Cyclin D1 and CDK2 (Fig. 3C), which were essential for transition of cell cycle from G1 to S phase. Flow cytometry showed that high-concentration PGD2 dose-dependently increased the cell apoptosis rates (Fig. 3D, E) in DLBCL cells. Notably, as the cleavage of caspase-3, caspase-9, and PARP is significant process in cell apoptosis, high-concentration PGD2 was observed to increase the expression of Bax, and the proportion of cleaved forms of caspase-3, caspase-9 and PARP (Fig. 3F), indicating the activation of apoptosis process in DLBCL cells.

Transwell assays showed the significant reduction of DLBCL cell crossing the transwell chamber membrane after the treatment with high-concentration PGD2 (Fig. 3G). Furthermore, the expression level of zeb1 and vimentin was also decreased by high-concentration PGD2 in DLBCL cells (Fig. 3H), which were important positive factors in cell invasion. Taken together, our findings suggested that high-concentration PGD2 could inhibit the progression of DLBCL through regulating cell proliferation, cell cycle, apoptosis, and invasion.

### High-concentration PGD2 induced ROS-mediated DNA damage and enhanced drug cytotoxicity in DLBCL cells

Recently, DNA damage signaling has been found to be involved in the development of various cancers [28], including pancreatic cancer [29–32], gastric cancer [33], myeloproliferative neoplasms [34], genitourinary cancer [35], ovarian cancer [36], breast cancer [37], and lymphoma [38]. 15d-PGJ2, the final metabolite of PGD2 in cells, was demonstrated to induce the production of intracellular ROS of chronic myelogenous leukemia cells [15] and B lymphoma cells [16], which could result in the lesions of DNA base and strand. Moreover, our previous study [13] found that PTGDS, a key enzyme in PGD2 production, was closely associated with DNA damage in DLBCL. Therefore, we performed further experiments to explore the effects of high-concentration PGD2 on ROS accumulation and DNA damage in DLBCL cells.

**Fig. 4:**
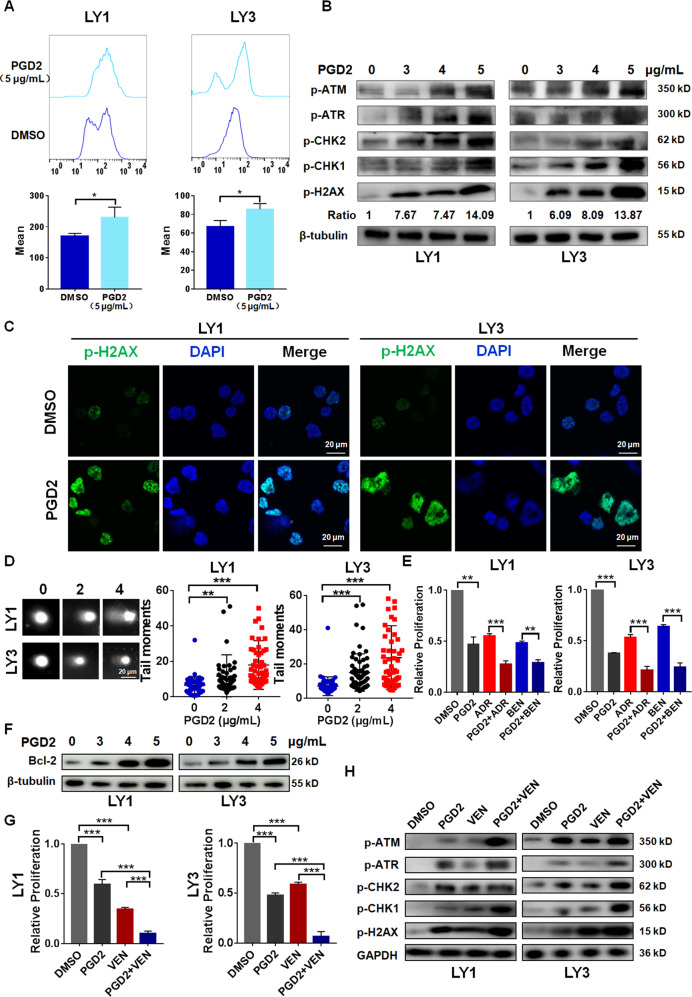
High-concentration PGD2 induced ROS-mediated DNA damage and enhanced drug cytotoxicity in DLBCL cells. **A** High-concentration PGD2 increased the mean fluorescence intensity of DLBCL cells loaded with DCFH-DA probe, indicating the intracellular accumulation of ROS. **B** High-concentration PGD2 promoted the phosphorylation of key regulators in DNA damage pathway. **C** Immunofluorescent images indicated that high-concentration PGD2 increased the expression of p-H2AX in DLBCL cells. Bar = 20 μm. **D** Representative images and quantification of the tail in comet assay. Bar = 20 μm. **E** High-concentration PGD2 enhanced the cytotoxicity of Adriamycin (ADR) and Bendamustine (BEN) in LY1 and LY3 cells. **F** High-concentration PGD2 increased the expression of Bcl-2 in DLBCL cells. **G**, **H** High-concentration PGD2 was observed to enhance the cytotoxicity of venetoclax in DLBCL cells. Cells were treated with drugs for 48 h. Data are shown as the mean ± SD. *p < 0.05; **p < 0.01; ***p < 0.001.

High-concentration PGD2 was found to induce the intracellular accumulation of ROS in DLBCL cells (Fig. 4A). Results of western blotting and immunofluorescence assays showed that high-concentration PGD2 dose-dependently increased the phosphorylation of key regulators in DNA damage pathway (Fig. 4B, C), such as ATM, ATR, CHK1, CHK2, H2AX, indicating the increased activation of DNA damage pathway. Furthermore, comet assay demonstrated that DLBCL cells treated with high-concentration PGD2 showed longer tail moment (Fig. 4D), suggesting the increase in DNA damage. Taken together, our results indicated that high-concentration PGD2 might enhance DNA damage through inducing intracellular ROS accumulation in DLBCL cells.

Further experiments were performed to investigate the effects of PGD2 on drug cytotoxicity of DNA damaging drugs, adriamycin and bendamustine [39, 40] in DLBCL cells. Our findings observed that high-concentration PGD2 enhanced the anti-tumor activity of adriamycin and bendamustine in DLBCL (Fig. 4E), indicating the potential role of high-concentration PGD2 in combination chemotherapy for DLBCL treatment.

Our investigation showed that high-concentration PGD2 induced cell apoptosis, but significantly increased the expression of anti-apoptotic factor Bcl-2 in DLBCL cells (Fig. 4F). Besides, Bcl-2 has been found to resist to cell death [41] through promoting the recovery from DNA damage [42, 43], suggesting the potential role of Bcl-2 inhibition in tumor treatment through DNA damage. Therefore, we performed further experiments to explore the role of high-concentration PGD2 in drug response to venetoclax, which was the inhibitor of Bcl-2 and displayed greatly anti-tumor effects in hematological cancers. High-concentration PGD2 was observed to enhance the cytotoxicity of venetoclax in DLBCL (Fig. 4G). Furthermore, the combination application of PGD2 and venetoclax displayed better efficiency on the activation of DNA damage signaling than single administration (Fig. 4H).

Collectively, these findings provided evidence that high-concentration PGD2 might sensitize DLBCL cells to DNA damaging drugs and venetoclax through inducing ROS-dependent DNA damage. Further in vitro and in vivo studies were needed to illuminate the role and molecular mechanism of high-concentration PGD2 in combined therapy for DLBCL treatment.

### HDAC inhibitors regulated the expression and effects of PGD2 and CRTH2 in DLBCL cells

**Fig. 5:**
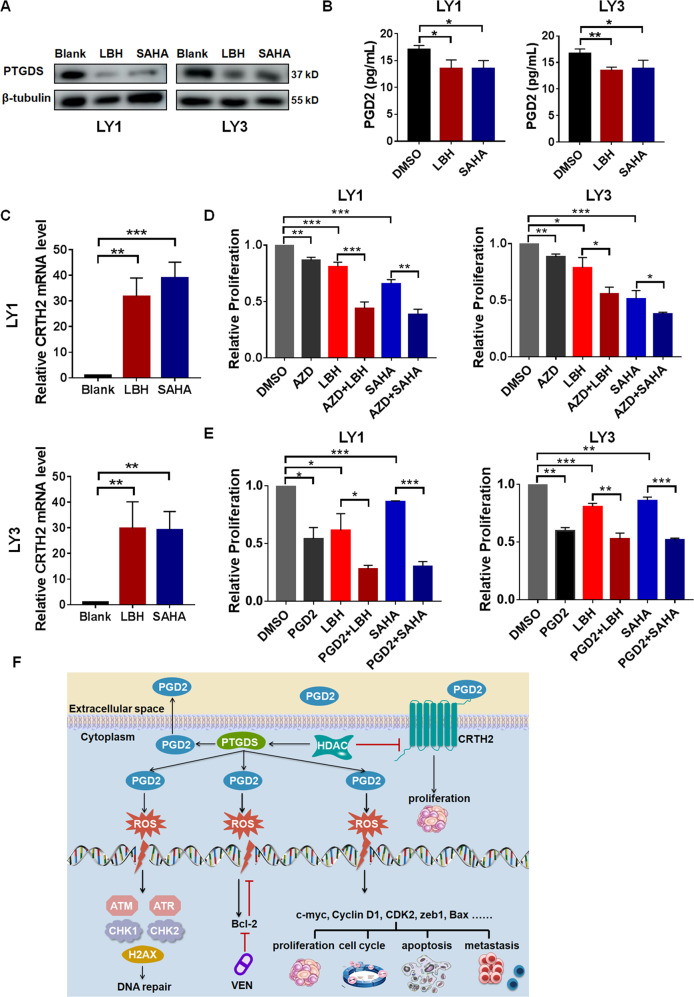
HDAC inhibitors regulated the expression and effects of PGD2 and CRTH2 in DLBCL cells. **A**, **B** The expression level of PTGDS and the production of PGD2 were decreased by vorinostat (SAHA) and panobinostat (LBH589) in DLBCL cells. **C** SAHA and LBH589 increased the expression of CRTH2 in DLBCL cells. The expression of CRTH2 in cells without drug treatment was adjusted to 1. **D**, **E** The treatment of AZD1981 (AZD) and high-concentration PGD2 enhanced the cytotoxicity of HDAC inhibitors in LY1 and LY3 cells. **F** Mechanism diagram summarized that PGD2-CRTH2 enhanced tumor progression and high-concentration PGD2 exhibited potent anti-tumor activities through ROS-dependent DNA damage in DLBCL. Cells were treated with drugs for 48 h. Data are shown as the mean ± SD. *p < 0.05; **p < 0.01; ***p < 0.001.

Epigenetic modifications have been involved in the progression and therapy of several tumors [44], including lymphoma [45], and HDAC inhibitors, SAHA and LBH589 display anti-tumor effects in lymphoma treatment [46, 47]. We performed experiments to explore whether acetylation modification have influence on the effects of PGD2 in DLBCL. It’s found that SAHA and LBH589 decreased the expression of PTGDS and the production of PGD2 in DLBCL cells (Fig. 5A, B), which might inhibit the oncogenic role of low-concentration PGD2. As both of them increased the expression level of CRTH2 in DLBCL cells (Fig. 5C), further experiments were performed to elucidate the effects of combination therapy between HDAC inhibitors and CRTH2 inhibitor in DLBCL. Notably, the addition of AZD1981 enhanced the cytotoxicity of HDAC inhibitors in terms of cell proliferation (Fig. 5D). Furthermore, high-concentration PGD2 could also enhance the anti-tumor effects of HDAC inhibitors in DLBCL (Fig. 5E). Taken together, these findings provided a basis for new therapeutic strategies for DLBCL patients, and detailed molecular mechanisms involved in the combined therapy needs further experimental investigation.

## Discussion

In this study, our investigations elucidated for the first time the expression level, regulatory role and molecule mechanism of PGD2 in DLBCL development. The increased concentration of serum PGD2 and decreased expression of CRTH2 were associated with clinical characteristics and prognosis in DLBCL patients. Interestingly, PGD2 with different concentration exerted divergent effects on DLBCL progression. Low-concentration PGD2 promoted the proliferation of DLBCL cells while high-concentration PGD2 exhibited potent therapeutic potential through regulating cell proliferation, apoptosis, cycle and invasion. Moreover, high-concentration PGD2 could enhance the cytotoxicity of DNA damage drugs, adriamycin and bendamustine, and Bcl-2 inhibitor venetoclax in DLBCL cells through inducing ROS-mediated DNA damage. HDAC inhibitors, SAHA and LBH589, were found to regulate PGD2 production and CRTH2 expression, and high-concentration PGD2 and CRTH2 inhibitor AZD1981 enhanced their cytotoxicity in DLBCL. These results provided theoretical basis for the application of novel prognostic markers and therapeutic strategies in the treatment of DLBCL.

Previous studies about PGD2 have focused on its effects in the development of inflammation [48], parasitic infections [49], and respiratory disease [50]. However, few studies reported the expression level of PGD2 in disease patients, including DLBCL. Our previous study demonstrated the high expression of PTGDS, one of PGD2 synthetase, in DLBCL cells and patients [13]. Consistently, the present study observed high concentration of serum PGD2 in DLBCL patients. In previous researches, CRTH2, the receptor of PGD2 was found to be highly expressed in gastric cancer patients [51] and lowly expressed in classical Hodgkin’s lymphoma [52]. In our study, the decreased expression of CRTH2 was observed in DLBCL cell lines and tumor tissues. Furthermore, high PGD2 concentration and decreased CRTH2 expression were closely correlated with clinical features, therapeutic effects and prognosis of DLBCL patients. Therefore, these findings indicated that PGD2 and CRTH2 might be potential diagnostic biomarkers and prognostic predictors in DLBCL patients, and further interrogations with more enrolled patients are warranted to confirm the prognostic role of PGD2 and CRTH2 in DLBCL.

The anti-proliferation effects of PGD2 in tumors have been demonstrated in previous studies, including gastric cancer, lung cancer, melanoma and colon cancer [10, 11, 27, 53]. In addition, previous researchers showed that PGD2 exerted anti-tumor potency through increasing apoptosis [54], inducing G0/G1 cell cycle arrest [55] and depleting metastasis [55–57]. Consistently, our study found that high-concentration PGD2 could inhibit the progression of DLBCL through regulating multiple biological functions, including cell proliferation, cell cycle, cell apoptosis and cell invasion. These results provided evidence that high-concentration PGD2 had therapeutic potential against DLBCL. Interestingly, low-concentration PGD2, similar to serum PGD2 in DLBCL patients, displayed promoting effects in the proliferation of DLBCL cells through binding to CRTH2. Moreover, previous study found that tumor-derived PGD2 could activate myeloid-derived suppressor cells and support tumor growth [58]. These results might partly explain the pro-tumor effects of serum PGD2 and further detailed investigations are needed to illuminate the mechanism of PGD2-CRTH2 in the development of DLBCL.

Previous investigations have implicated that multiple molecules and pathways were involved in the effects of PGD2 in tumor progression. For example, in acute promyelocytic leukemia, tumor-derived PGD2 bound and stimulated the CRTH2 on innate lymphoid cells, activated monocytic myeloid-derived suppressor cells, and ultimately promoted tumor progression [58]. The interaction between PGD2 and its receptor could regulate cAMP production [59] and SOX9 expression [60], and then have influence on tumor development. Besides, the regulatory role of PGD2 on PPARγ [10, 14] activation and STAT3 phosphorylation [61] was demonstrated in the development of gastric cancer and prostate tumor. PGD2 has been involved in the production of ROS [15, 16]. In this study, we demonstrated that high-concentration PGD2 exerted anti-tumor effects through inducing ROS accumulation and then DNA damage in DLBCL. Furthermore, it’s shown that high-concentration PGD2 enhanced the cytotoxic effects of DNA damage drugs in DLBCL cells. These results provided evidence for the potential of high-concentration PGD2 in DLBCL treatment, and further in vivo and preclinical studies were needed to promote its clinical application.

Epigenetic modification play significant roles in tumor development, and targeting epigenetic changes are recognized as promising therapeutic strategy for tumor therapy, especially in hematological malignancies [62]. Multiple kinds of epigenetic modifying drugs have been approved by FDA for lymphoma therapy, such as HDAC inhibitors and DNMT inhibitors [63]. However, the application of epigenetic modifying drugs in tumor therapy is somewhat hampered because of lacking detailed understanding of its selectivity and mechanisms. Previous studies found that HDAC inhibitors could regulate the release of PGD2 [25] and the activation of DNA damage signaling [64]. In our study, HDAC inhibitors decreased the expression of PTGDS and then the production of PGD2 in DLBCL cells, which might impede the proliferation promoting effects of serum PGD2. Furthermore, abnormal methylation has also been involved in PGD2 production [65] and DNA damage pathway [66]. Therefore, further basic and clinical studies are warranted to evaluate the therapeutic effect of combined strategies between PGD2 and epigenetic drugs in tumor therapy.

In summary, our results demonstrated for the first time the high concentration of serum PGD2 and decreased expression of CRTH2, and their clinical correlation in DLBCL patients. PGD2 with different concentration exerted divergent effects on DLBCL progression. Low-concentration PGD2 promoted the growth of DLBCL through binding to its receptor CRTH2. Notably, high-concentration PGD2 displayed excellent anti-lymphoma effects via inducing ROS-mediated DNA damage. Besides, HDAC inhibitors influenced the expression and effects of PGD2-CRTH2, and AZD1981 and high-concentration PGD2 could enhance their anti-tumor effects in DLBCL. Collectively, our findings demonstrated PGD2 and CRTH2 as novel prognostic biomarkers and therapeutic targets in DLBCL, and highlighted the potency of high-concentration PGD2 as a promising therapeutic strategy for DLBCL patients.

## Materials and methods

### Clinical specimens and cell lines

This study was approved by the Medical Ethical Committee of Shandong Provincial Hospital, and written informed consent from each patient and volunteer was conformed to the Declaration of Helsinki. Histological diagnoses in accordance with the 2016 WHO classification were established [67]. Serum and peripheral blood mononuclear cells (PBMCs) were isolated from the whole blood of DLBCL patients and healthy donors from 2017 to 2019. CD19^+^ B cells were purified from freshly isolated PBMCs of healthy donors. The clinical information was collected in the database of Shandong Provincial Hospital. LY1, LY3, LY8, LY10, VAL, U2932, SU-DHL-2 cells were bought from ATCC, cultured in Iscove modified Dulbecco medium (IMDM, Gibco, CA, USA) enriched with 10% heat-inactivated fetal bovine serum (HyClone, UT, USA), 1% penicillin/streptomycin mixture and 2 mM glutamine, and incubated at 37 °C and 5% CO_2_. All cells were periodically examined for mycoplasma infection and STR (Short Tandem Repeat).

### Reagents

PGD2 was bought from Cayman Chemical (12010, MI, USA) and AZD1981 was from MCE (HY-15950, NJ, USA). Adriamycin (ADR) and bendamustine (BEN) were purchased from Selleck Chemicals (TX, USA). Venetoclax (VEN) was from MCE (HY-15531, USA). SAHA (SML0061) and LBH589 (SML3060) were purchased from Sigma-Aldrich (MO, USA).

### In silico analysis

Microarray datasets of GSE31312, GSE56315, and GSE57611 were downloaded from the GEO database (www.ncbi.nlm.nih.gov/geo). The expression level of CRTH2 in DLBCL patients was evaluated based on GSE56315. The association between CRTH2 expression and clinical characteristics was assessed using data from GSE31312. The Kaplan–Meier survival curves were generated to explore the prognostic role of CRTH2 in DLBCL patients and the optimal cutoff was selected by scan model. Gene ontology (GO) analysis was performed based on GSE31312 and GSE57611. The immunohistochemical pictures of CRTH2 in lymphoma tissue were from The Human Protein Atlas database (https://www.proteinatlas.org/).

### Elisa assay

Peripheral blood from 53 DLBCL patients and 19 healthy volunteers was collected and then serum was isolated by centrifugation. DLBCL cells with indicated treatment were cultured and the supernatant was collected. The concentration of PGD2 was examined with a commercial ELISA kit (MB-4041, MBBIOLOGY, China) according to the manufacturer’s protocol.

### Quantitative real-time PCR

Total RNA was extracted using RNAiso Plus reagent (Takara, Dalian, China) and the synthesis of cDNA library was performed using PrimeScript RT reagent kit with gDNA eraser (Takara). Relative mRNA levels were finally detected by SYBR Green Master Mix (TaKaRa) in LightCycler 480II real-time PCR system (Roche, Basel, Swizerland). GAPDH was used as internal reference. The CRTH2 primers were as follows: forward, 5′-CACTGCCCAAAGTGCTTCCA-3′; reverse, 5′-TGCTGTGCCCATTCAACTTCTAAC-3′. The quantitative RT-PCR assay was biologically repeated for three times. The relative expressional level was finally calculated using the standard 2-ΔΔCT method.

### Western blotting

Total protein extraction and western blotting were performed following standard methods [68]. Equivalent protein (30 μg) of each group was electrophoresed. The primary antibodies included PTGDS (ab182141, Abcam) and other antibodies bought from Cell Signaling Technology (Cell Signaling Technology, Beverly, USA), including c-myc (18583), Cyclin D1 (2922), CDK2 (2546), caspase 3 (9662), caspase 9 (9508), PARP (9532), Bax (5023), zeb-1(3396), vimentin (5741), p-ATM (Ser1981, 5883), p-ATR (Ser428, 2853), p-CHK1 (Ser345, 2348), p-CHK2 (Thr68, 2197), p-H2AX (Ser139, 9718), and Bcl-2 (15071). β-tubulin (86298) and GAPDH (97166) were served as the internal reference.

### Cell proliferation and invasion assay

Cell Counting Kit-8 (CCK-8) kits (CK04, Dojindo, Japan) and Multiskan GO Microplate Reader (Thermo Scientific, IL, USA) were used as previously described [69] to evaluate the proliferation level of DLBCL cells. The proliferation of cells treated with DMSO was adjusted to 1. Cell invasion was assessed using 24-well transwell chambers (8.0 μm, Corning, USA) precoated with matrigel.

### Flow cytometry analysis

Flow cytometry was performed to assess cell cycle and cell apoptosis. DLBCL cells with indicated treatment were collected from six-well plates and washed three times with pre-cooled PBS. In cell cycle assay, DLBCL cells should be fixed with 70% ethanol overnight at −20 °C. For staining, Propidium iodide (PI, 550825, BD Biosciences, MA, USA) was used in cell cycle analysis and Annexin V-FITC apoptosis detection kit (556547, BD Biosciences) was applied for cell apoptosis analysis. Stained cells were analyzed by Navios Flow Cytometer (Beckman Coulter, CA, USA). Data analyses were performed with FlowJo software.

### Measurement of ROS level

The DCFH-DA probe was used to detect the level of intracellular ROS. The ROS level in DLBCL cells was assessed by ROS Assay Kit (S0033, Beyotime, China) according to the manufacturers’ protocols.

### Immunofluorescence assays and confocal microscopy

DLBCL cells with indicated treatment were transferred to a glass slide using cytospin. 4% formaldehyde fixation was applied for 15 min and DLBCL cells were permeabilized using 0.1% Triton X 100 for 10 min After blocked with 5% goat serum for 1 h, slides were incubated with the primary antibody (p-H2AX, Ser139, 9718, CST) at 4 °C overnight. Slides were further incubated with secondary antibodies and DAPI. Leica TCS SP8 MP confocal microscope system (Germany) was used for confocal microscopy.

### Comet assay

To detect the breaks of DNA, alkaline comet assays were performed using the single-cell gel electrophoresis assay kit (4250-050-K, Trevigen) according to the protocol.

### Statistical analysis

All in vitro experiments were performed in triplicate and results were presented as mean ± standard deviation (SD) of data obtained from three separate experiments. Data were tested for homogeneity of variances and normality. Statistical analysis of quantitative variables was performed using Students *t*-test and non-parametric tests. Survival curves were calculated by Kaplan–Meier method and the differences between two groups were compared by log-rank test. Chi-square test was used to analyze the correlation between clinical parameters and serum PGD2 concentration in DLBCL patients. There was no statistical method used to determine the sample size in our study. All calculations were made in SPSS version 23.0 software (SPSS Inc., IL, USA). The differences were considered statistically significant at *p* < 0.05 (*p < 0.05, **p < 0.01, ***p < 0.001).

The detailed information is described in Supplementary Materials and Methods.

## Supplementary information

Supplementary Materials and Methods

Supplementary Figures

Supplementary Table 1

Supplementary Table 2

Original Data File

## Data Availability

The datasets used and/or analyzed during the current study are available from the corresponding authors on reasonable request.
