# Supplementary material for: PGD2 displays distinct effects in diffuse large B-cell lymphoma depending on different concentrations
Source: Cell Death Discov. 2023 Feb 1;9:39. doi: 10.1038/s41420-023-01311-6 (PMC9892043; doi:10.1038/s41420-023-01311-6)
Supplement: Supplementary file 1 — Supplementary Materials and Methods [file 41420_2023_1311_MOESM1_ESM.docx]

**PGD2 displays distinct effects in diffuse large B-cell lymphoma depending on different concentrations**

Shunfeng Hu^1,2^,Tiange Lu^1^, Juanjuan Shang^1^, Yiqing Cai^1^, Mengfei Ding^1^, Xiangxiang Zhou^2,3,4*^, Xin Wang^1,2,3,4*^

^1^ *Department of Hematology, Shandong Provincial Hospital, Shandong University, Jinan, Shandong, 250021, China.*

^2^ *Department of Hematology, Shandong Provincial Hospital Affiliated to Shandong First Medical University, Jinan, Shandong, 250021, China.*

^3^ *Branch of National Clinical Research Center for Hematologic Diseases, Jinan, Shandong, 250021, China.*

^4^ *National Clinical Research Center for Hematologic Diseases, the First Affiliated Hospital of Soochow University, Suzhou, 251006, China.*

**Running title:** PGD2 in the development of DLBCL

^*^**Corresponding authors**

Xin Wang, M.D., Ph.D.

Director & Professor of Department of Hematology,

Shandong Provincial Hospital, Shandong University

Add: No.324, Jingwu Road, Jinan, Shandong, 250021, China

Tel: 0086-531-68776358(B); 0086-13156012606(M)

Fax: 0086-531-87061197(B);

Email: [xinw007@126.com](mailto:xinw007@126.com)

Xiangxiang Zhou, M.D., Ph.D.

Department of Hematology, Shandong Provincial Hospital Affiliated to Shandong First Medical University

Add: No.324, Jingwu Road, Jinan, Shandong, 250021, China.

Tel: 0086-531-68776358(B); 0086-15866695595(M)

E-mail: [xiangxiangzhou@sdu.edu.cn](mailto:xiangxiangzhou@sdu.edu.cn)

**Conflict of Interest Disclosures**

The authors declare no competing financial interests.

Supplementary Materials and Methods

**Clinical specimens and cell lines**

This study was approved by the Medical Ethical Committee of Shandong Provincial Hospital and written informed consent from each patient and volunteer was conformed to the Declaration of Helsinki. Histological diagnoses in accordance with the 2016 WHO classification were established. Serum and peripheral blood mononuclear cells (PBMCs) were isolated from the whole blood of DLBCL patients and healthy donors from 2017 to 2019. CD19^+^ B cells were purified from freshly isolated PBMCs of healthy donors. The clinical information, including demographic information, International Prognostic Index (IPI) score, stage, routine blood tests, image examination, bone marrow tests and immunohistochemistry (IHC) results, was collected in the database of Shandong Provincial Hospital. LY1, LY3, LY8, LY10, VAL, U2932, SU-DHL-2 cells were bought from ATCC, cultured in Iscove modified Dulbecco medium (IMDM, Gibco, CA, USA) enriched with 10% heat-inactivated fetal bovine serum (HyClone, UT, USA), 1% penicillin/streptomycin mixture and 2 mM glutamine, and incubated at 37 °C and 5% CO_2_. All cells were periodically examined for mycoplasma infection and STR (Short Tandem Repeat).

**Reagents**

PGD2 was bought from Cayman Chemical (12010, MI, USA) and AZD1981 was from MCE (HY-15950, NJ, USA). Adriamycin (ADR) and bendamustine (BEN) were purchased from Selleck Chemicals (TX, USA). Venetoclax (VEN) was from MCE (HY-15531, USA). SAHA (SML0061) and LBH589 (SML3060) were purchased from Sigma-Aldrich (MO, USA).

**In silico analysis**

Microarray datasets of GSE31312, GSE56315 and GSE57611 were downloaded from the GEO database ([www.ncbi.nlm.nih.gov/geo](http://www.ncbi.nlm.nih.gov/geo)). The expression level of CRTH2 in DLBCL patients was evaluated based on GSE56315. The association between CRTH2 expression and clinical characteristics was assessed using data from GSE31312. The Kaplan-Meier survival curves were generated to explore the prognostic role of CRTH2 in DLBCL patients and the optimal cutoff was selected by scan model. Gene ontology (GO) analysis was performed based on GSE31312 and GSE57611. The immunohistochemical pictures of CRTH2 in lymphoma tissue were from The Human Protein Atlas database (<https://www.proteinatlas.org/> ).

**Elisa assay**

Peripheral blood from 53 DLBCL patients and 19 healthy volunteers was collected and then serum was isolated by centrifugation at 1,000×g for 15 minutes within 2 hours. DLBCL cells with indicated treatment were cultured and the supernatant was collected. Samples were stored at −80°C and centrifuged after thawing before use. The concentration of PGD2 in serum and cell culture supernatants was examined with a commercial ELISA kit (MB-4041, MBBIOLOGY, China) according to the manufacturer’s protocol.

**Quantitative real-time PCR**

Total RNA was extracted from DLBCL cells using RNAiso Plus reagent (Takara, Dalian, China) according to manufacturer’s instructions, as previous reported. The synthesis of cDNA library was performed using PrimeScript RT reagent kit with gDNA eraser (Takara). Relative mRNA levels were finally detected by SYBR Green Master Mix (TaKaRa) in LightCycler 480II real-time PCR system (Roche, Basel, Swizerland) as instructed by the manufacturer. GAPDH was used as internal reference. The CRTH2 primers were as follows: forward, 5′-CACTGCCCAAAGTGCTTCCA-3′; reverse, 5′-TGCTGTGCCCATTCAACTTCTAAC-3′. The quantitative RT-PCR assay was biologically repeated for three times. The relative expressional level was finally calculated using the standard 2-ΔΔCT method.

**Western blotting**

To collect total protein, DLBCL cells were washed three times with pre-cooled PBS, and then lysed on ice for 30 minutes using lysis buffer (ShenergyBiobolor, Shanghai, China) with PMSF and 1×phosphatase inhibitor cocktail (PhosSTOP, Roche, Basel, Switzerland). The concentration of protein was measured by BCA assay (Shenergy Biocolor). Then, equivalent protein (30 μg) of each group was electrophoresed on 7.5%-12% SDS-PAGE gels and transferred to PVDF membrane (Millipore, MA, USA). After the incubation with 5% skim milk in TBS with 0.1% tween-20 (TBST) for 1 hour at room temperature, membranes were incubated with primary antibodies at 4℃ overnight. After washed with TBST three times, membranes were incubated with corresponding HRP-conjugated secondary antibodies (Zhongshan Goldenbridge). After treatment with chemiluminescence reagent system (Merck Millipore, MA, USA), chemiluminescent signals were detected by the Amersham Imager 600 imaging system (General Electric, USA). ImageJ software (NIH) was used to quantify the protein bands normalized to control，as previous reported [27]. The primary antibodies included PTGDS (ab182141, Abcam) and other antibodies bought from Cell Signaling Technology (Cell Signaling Technology, Beverly, USA), including c-myc (18583), Cyclin D1 (2922), CDK2 (2546), caspase 3 (9662), caspase 9 (9508), PARP (9532), Bax (5023), zeb-1(3396), vimentin (5741), p-ATM (Ser1981, 5883), p-ATR (Ser428, 2853), p-CHK1 (Ser345, 2348), p-CHK2 (Thr68, 2197), p-H2AX (Ser139, 9718), and Bcl-2 (15071). β-tubulin (86298) and GAPDH (97166) were served as the internal reference.

**Cell proliferation assay**

Cell Counting Kit-8 (CCK-8) assays were performed as previously described [28] to evaluate the proliferation level of DLBCL cells with indicated treatment. DLBCL cells (1×10^4^ cells/ 100μl/ well) were seeded into 96-well plates with or without indicated drugs and cultured for 24-96 hours. Then, 10 μL CCK-8 kit (CK04, Dojindo, Japan) was added into 96-well plates and incubated for 1-4 hous at 37°C according to the manufacturer’s proposal. The light absorption at 450 nm was detected using Multiskan GO Microplate Reader (Thermo Scientific, IL, USA). The proliferation of cells treated with DMSO was adjusted to 1.

**Cell invasion assay**

Cell invasion analysis of DLBCL cells was conducted using 24-well transwell chambers (8.0 μm, Corning, USA) precoated with matrigel. Briefly, 1×10^5^ DLBCL cells in 200 μL serum-free IMDM were seeded into the upper chamber and 600 μL IMDM with 10% FBS was added to the lower chamber. After the incubation at 37 ℃ with 5% CO_2_ for 24-48 hours, the number of DLBCL cells in lower chamber was counted using cell counting plate.

**Flow cytometry analysis**

Flow cytometry was performed to assess cell cycle and cell apoptosis. DLBCL cells with indicated treatment were collected from six-well plates and washed three times with pre-cooled PBS. In cell cycle assay, DLBCL cells should be fixed with 70% ethanol overnight at -20 ℃. For staining, Propidium iodide (PI, 550825, BD Biosciences, MA, USA) was used in cell cycle analysis and Annexin V-FITC apoptosis detection kit (556547, BD Biosciences) was applied for cell apoptosis analysis. Stained cells were analyzed by Navios Flow Cytometer (Beckman Coulter, CA, USA). Data analyses were performed with FlowJo software.

**Measurement of ROS level**

The DCFH-DA probe was used to detect the level of intracellular ROS. The ROS level in DLBCL cells was assessed by ROS Assay Kit (S0033, Beyotime, China) according to the manufacturers’ protocols. Briefly, DLBCL cells with indicated treatment were collected from six-well plates and washed three times with pre-cooled PBS. DLBCL cells were incubated with 10 µM DCFH-DA and protected from lightat 37 °C for 30 minutes. According to the instructions, ROS levels were analyzed by Navios Flow Cytometer (Beckman Coulter, CA, USA).

**Immunofluorescence assays and confocal microscopy**

DLBCL cells with indicated treatment were transferred to a glass slide using cytospin. 4% formaldehyde fixation was applied for 15 minutes and DLBCL cells were permeabilized using 0.1% Triton X 100 for 10 minutes. After blocked with 5% goat serum for 1 hour, slides were incubated with the primary antibody (p-H2AX, Ser139，9718, CST) at 4 °C overnight. Slides were further incubated with secondary antibodies and DAPI. Leica TCS SP8 MP confocal microscope system (Germany) was used for confocal microscopy.

**Comet assay**

To detect the breaks of DNA, alkaline comet assays were performed using the single-cell gel electrophoresis assay kit (4250-050-K, Trevigen) according to the protocol. 1×10^4^ DLBCL cells were seed into 96-well plates and treated with high-concentration PGD2. After 48-hour culture, DLBCL cells were harvested and washed three times with 1×PBS. 50 μL DLBCL cell suspension was mixed with 500 μL comet LMAgarose and then pipetted 50 µL on slides. After incubation at 4 °C for 30 minutes, slides were immersed in alkaline lysis buffer overnight and then incubated in alkaline unwinding solution for 40 minutes. Electrophoresis was performed for 30 minutes at 300 mA and 1 V/cm in ice-cold alkaline electrophoresis solution. DAPI was used to stain slides and images were acquired using Olympus (IX73) inverted microscope. The comets of DLBCL cells were analyzed using CASP software and tail moment was measured statistically in at least 50 cells per group.

**Statistical analysis**

All *in-vitro* experiments were performed in triplicate and results were presented as mean ± standard deviation (SD) of data obtained from three separate experiments. Data were tested for homogeneity of variances and normality. Statistical analysis of quantitative variables was performed using Students t-test and non-parametric tests. Progression-free survival (PFS) was defined from diagnosis to disease progression, death, or last follow-up. Overall survival (OS) was defined from diagnosis to death or last follow-up. Survival curves were calculated by Kaplan-Meier method and the differences between two groups were compared by log-rank test. Chi-square test was used to analyze the correlation between clinical parameters and serum PGD2 concentration in DLBCL patients. There was no statistical method used to determine the sample size in our study. All calculations were made in SPSS version 23.0 software (SPSS Inc, IL, USA). The differences were considered statistically significant at p < 0.05 (*p < 0.05, **p < 0.01, ***p < 0.001).
