# Supplementary material for: PGD2 displays distinct effects in diffuse large B-cell lymphoma depending on different concentrations
Source: Cell Death Discov. 2023 Feb 1;9:39. doi: 10.1038/s41420-023-01311-6 (PMC9892043; doi:10.1038/s41420-023-01311-6)
Supplement: Supplementary file 2 — Supplementary Figures [file 41420_2023_1311_MOESM2_ESM.docx]

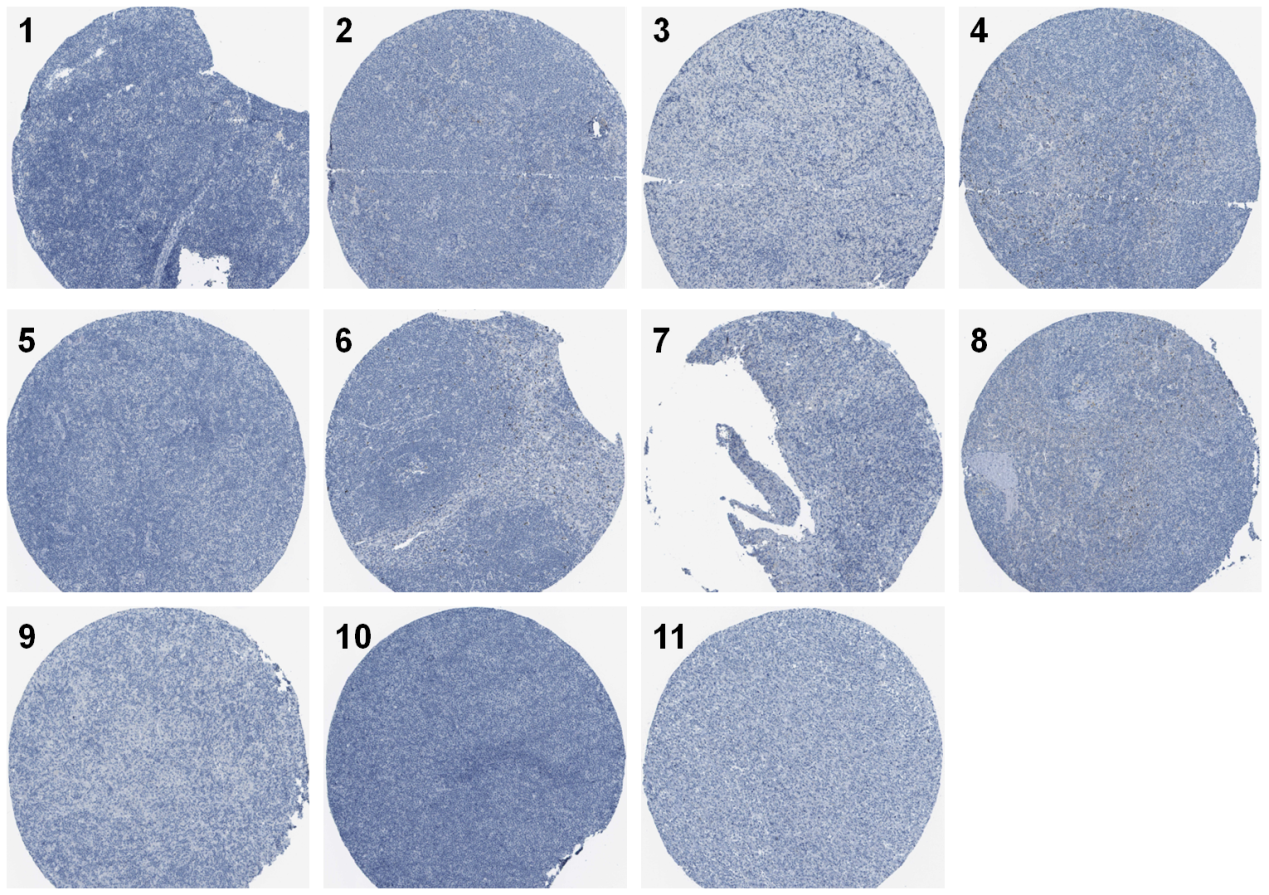


**Supplementary Figure 1**

The expression of CRTH2 in lymphoma tissue was negative, and immunohistochemical pictures were from The Human Protein Atlas database (https://www.proteinatlas.org/).


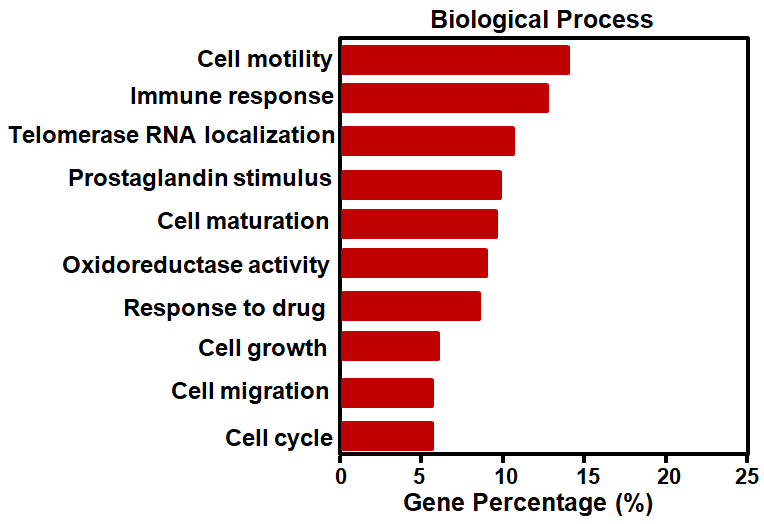


**Supplementary Figure 2**

Analysis of GSE57611 dataset confirmed the association between CRTH2 and DLBCL development.


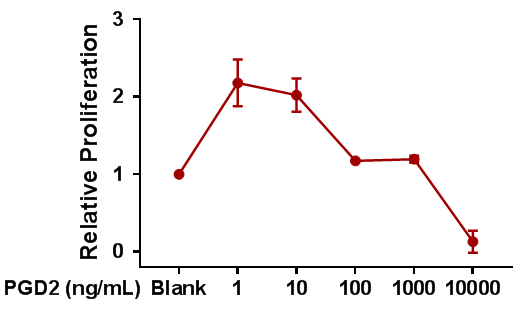


**Supplementary Figure 3**

CCK-8 assays with PGD2 range from 1-10000 ng/mL showed that low-concentration PGD2 (1-10 ng/mL) could promote the proliferation of DLBCL cells and high-concentration (1-10 μg/mL) significantly inhibited cell proliferation.
