# Supplementary material for: PGD2 displays distinct effects in diffuse large B-cell lymphoma depending on different concentrations
Source: Cell Death Discov. 2023 Feb 1;9:39. doi: 10.1038/s41420-023-01311-6 (PMC9892043; doi:10.1038/s41420-023-01311-6)
Supplement: Supplementary file 4 — Supplementary Table 2 [file 41420_2023_1311_MOESM4_ESM.docx]

**Supplementary Table 2.** Clinical characteristics based on PGD2 concentration in DLBCL patients.

| Characteristics | No. of  patients | Low PGD2  n (%) | High PGD2  n (%) | P value |
| --- | --- | --- | --- | --- |
| **Age(years)** |  |  |  |  |
| <60 | 30 | 24（45%） | 6（11%） | 0.549 |
| ≥60 | 23 | 19（36%） | 4（8%） |  |
| **Gender** |  |  |  |  |
| Male | 29 | 24（46%） | 5（9%） | 0.505 |
| Female | 24 | 19（36%） | 5（9%） |  |
| **Ann Arbor Stage** |  |  |  |  |
| Ⅰ/Ⅱ | 18 | 14（27%） | 4（7%） | 0.479 |
| Ⅲ/Ⅳ | 34 | 28（54%） | 6（12%） |  |
| **IPI Score > 3** |  |  |  |  |
| No | 38 | 28（54%） | 10（19%） | **0.03** |
| Yes | 14 | 14（27%） | 0（0%） |  |
| **Subtype** |  |  |  |  |
| GCB | 17 | 11（25%） | 6（13%） | **0.008** |
| Non-GCB | 28 | 27（60%） | 1（2%） |  |
| **B Symptoms** |  |  |  |  |
| Yes | 14 | 14（26%） | 0（0 %） | **0.033** |
| No | 39 | 29（55%） | 10（19%） |  |
| **Elevated** β**2-MG** |  |  |  |  |
| Yes | 19 | 16（30%） | 3（6%） | 0.484 |
| No | 34 | 27（51%） | 7（13%） |  |
| **Elevated** **LDH** |  |  |  |  |
| Yes | 28 | 24（45%） | 4（8%） | 0.291 |
| No | 25 | 19（36%） | 6（11%） |  |
| **LMR** |  |  |  |  |
| <3 | 39 | 29（55%） | 10（19%） | **0.033** |
| ≥3 | 14 | 14（26%） | 0（0%） |  |
| **Liver Invasion** |  |  |  |  |
| Yes | 2 | 1（2%） | 1（2%） | 0.345 |
| No | 51 | 42（79%） | 9（17%） |  |
| **Spleen Invasion** |  |  |  |  |
| Yes | 5 | 3（6%） | 2（4%） | 0.235 |
| No | 48 | 40（75%） | 8（15%） |  |
| **Double Expression** |  |  |  |  |
| Yes | 9 | 7（17%） | 2（5%） | 0.573 |
| No | 32 | 26（63%） | 6（15%） |  |
| **Therapeutic Efficacy** |  |  |  |  |
| CR+PR | 29 | 23（51%） | 6（13%） | 0.400 |
| SD+PD | 16 | 14（31%） | 2（5%） |  |

Abbreviations: IPI, international prognostic index; MG, microglobulin; LDH, lactate dehydrogenase; LMR, lymphocyte–monocyte ratio; CR, complete remission; PR, partial remission; SD, stable disease; PD, progressive disease.
