# Supplementary figures and images for: PGD2 displays distinct effects in diffuse large B-cell lymphoma depending on different concentrations
Source: Cell Death Discov. 2023 Feb 1;9:39. doi: 10.1038/s41420-023-01311-6 (PMC9892043; doi:10.1038/s41420-023-01311-6)

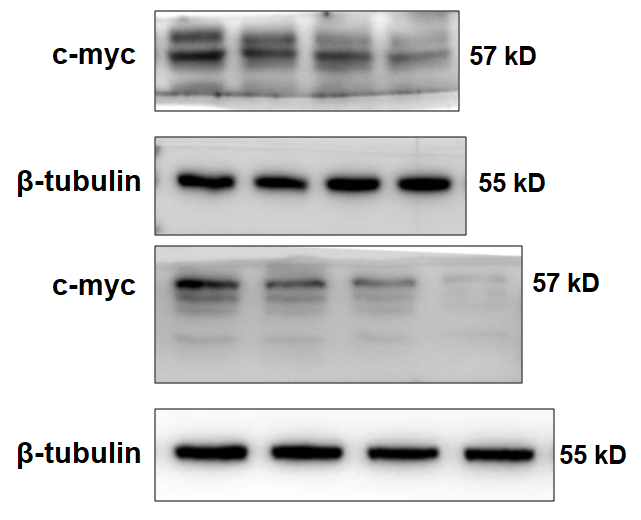


**Figure 2D**


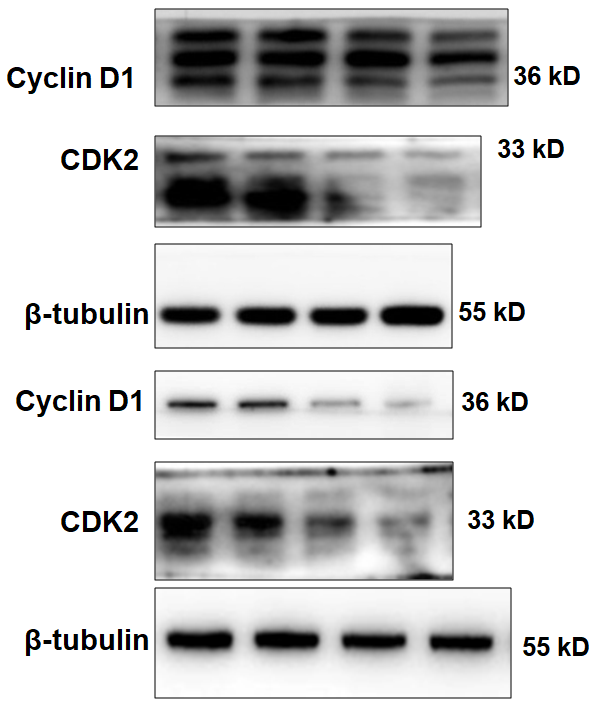


**Figure 3C**


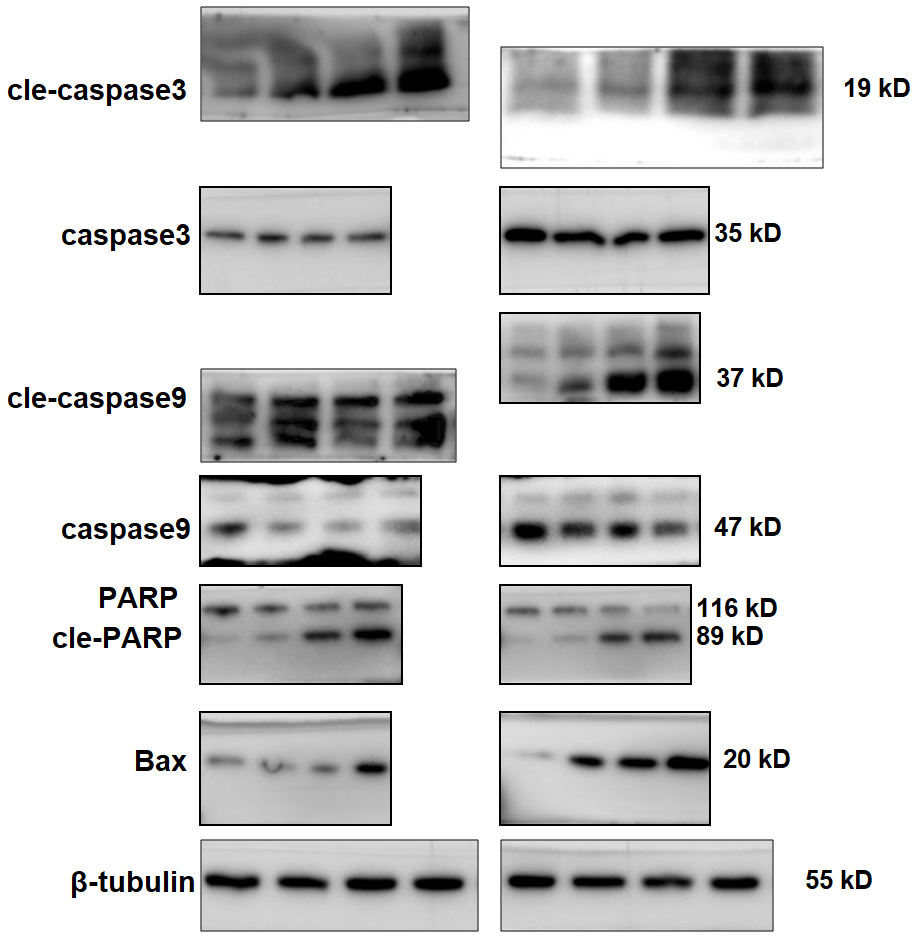


**Figure 3F**


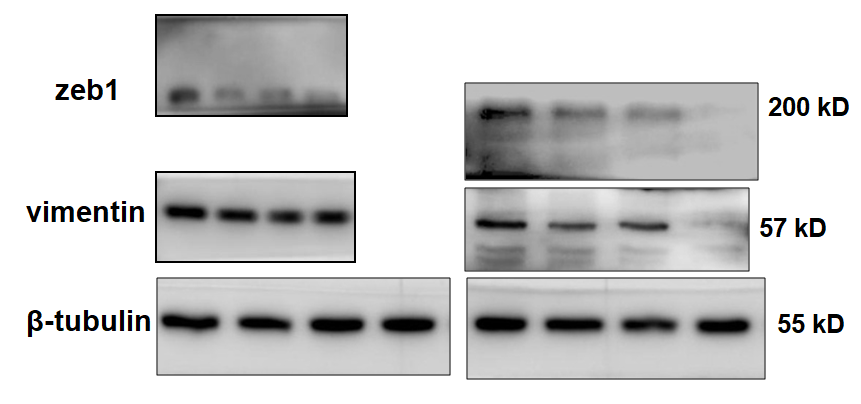


**Figure 3H**


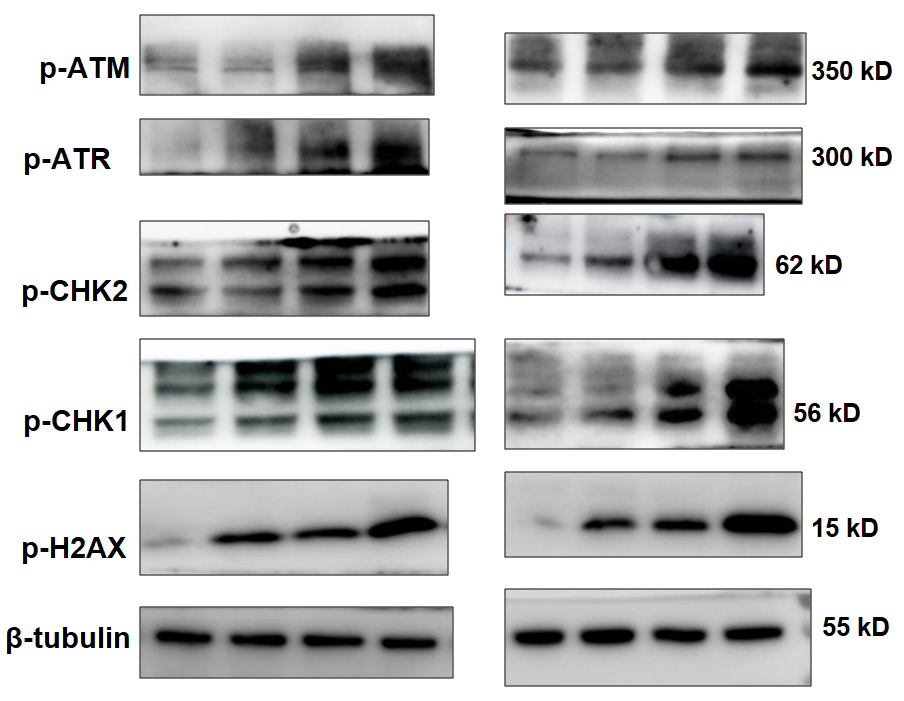


**Figure 4B**


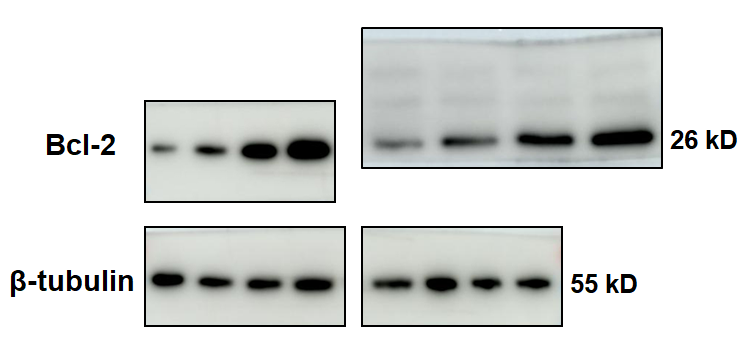


**Figure 4F**


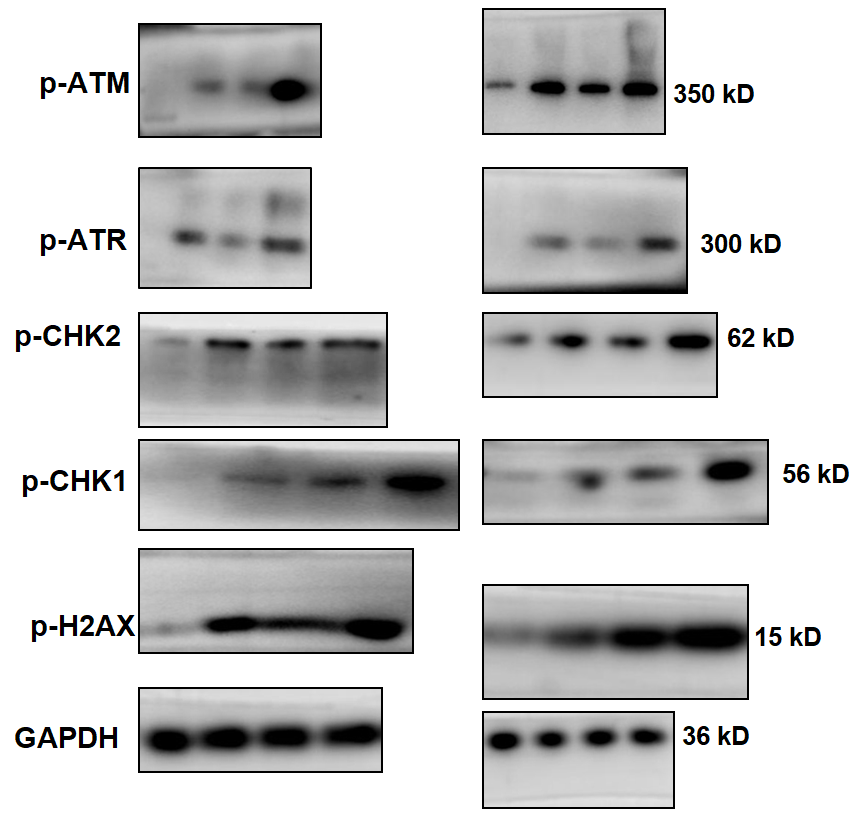
**Figure 4 H**


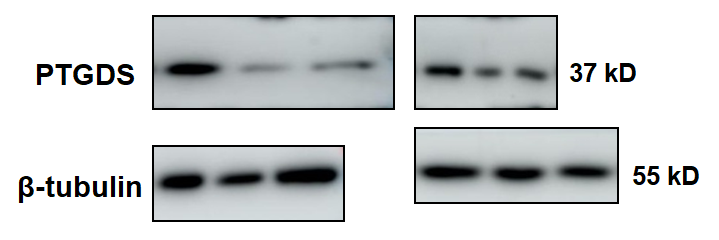


**Figure 5 A**

Supplement: Supplementary file 5 — Original Data File [file 41420_2023_1311_MOESM5_ESM.docx]
